# Glances at the Hospitals

**Published:** 1902-10-25

**Authors:** 


### GLANCES AT THE HOSPITALS.

### ROYAL DEVON AND EXETER HOSPITAL.

The total ordinary income was £6,338, and the total ordinary expenditure was £9,099. Legacies to the amount of £8,390 were received and also a donation of £52 10s. These brought the total receipts up to £14,823. Of the amount received in legacies, £3,000 was placed on deposit and the rest went to the general account. We do not see the cost per patient nor the cost per bed occupied anywhere stated. The medical and surgical tables are very meagre. They merely give the names of the diseases and the number of patients afflicted, and no results appear. There were 10 cases of acute rheumatism, 15 of acute pneumonia; but there is not even a mortality table from which we might obtain the results. So on all through the diseases mentioned. Even the operation table is not an exception. The operation for the radical cure of hernia was performed 16 times, herniotomy 7 times, and excision of the appendix once; but no further information is supplied.

### GENERAL HOSPITAL, BIRMINGHAM.

The ordinary income of this hospital amounted to £18,475, being an increase of £1,700 on that of the previous year; and the extraordinary income amounted to £8,205, being a decrease of £900 on that of the previous year. The ordinary expenditure was £24,411, and the extraordinary expenditure was £812, while £276 was transferred to the Jaffray branch of the hospital. On the previous year there was a deficit of £6,291, and this year there was a surplus of £1,180, which was applied to reduce the adverse balance, and which now stands at £5,110. We are glad to notice that the workpeople's own subscriptions amounted to £1,056. As regards the adverse balance the board "urgently appeals for new and increased subscriptions, and for donations." The average cost of each in-patient was £3 10s., the daily average number of patients resident in the hospital was 278, the average residence of each patient was 23 days, and the cost per annum of each bed occupied was £85. The total number of in-patients during the year was 5,170, and that of the out-patients was 67,813. No medical or surgical statistics are given—a most extraordinary omission in a hospital which publishes its 122nd annual report.

### ROYAL INFIRMARY, DUNDEE.

The total income amounted to £11,062 and the ordinary expenditure to £11,411, to which must be added extraordinary expenditure to the amount of £937, making a total of £12,348. The excess of expenditure over income was £1,285. Mr. J. K. Caird has most generously offered to erect a building, on the pavilion system, for the treatment of cancer. It will be placed behind the present infirmary, and will contain 100 beds. Mr. Caird has not stopped even at this munificent bequest, for he suggests that part of the annexe be fitted up as a laboratory for the investigation of disease, and for this purpose he guarantees £1,000 a year for five years. It is therefore not without reason that the directors of the infirmary record "their high appreciation of such large-hearted liberality." At the beginning of the year the infirmary contained 215 patients, and 245 remained at the end of it. During the year 3,188 were treated to a conclusion. Of these, 2,686 were discharged cured or relieved; 218 not relieved, and 290 died. The daily average number resident was 228; the average residence of each patient was 23 days; and the percentage of deaths was 8·47, or, deducting 83 patients admitted moribund, 6·02 per cent. The cost per bed occupied was £49 17s. The Roentgen Ray apparatus has been in almost daily use; and 20 cases have been treated by electric light on a modified Finsen method. The daily average number of nurses employed was 50, and the average number of servants was 44. A good training school for nurses exists in this infirmary, and it is worthy of note that 287 applications were received during the year by those anxious to become nurses. The medical and surgical statistics are admirably arranged. The tables are divided into eight columns, and show every necessary particular. There were 73 cases of rheumatic fever. All of these recovered, and the average residence was 22½ days. Of 50 cases of diphtheria 7 died. Of 19 cases of typhoid fever 2 died, with an average residence of 61 days for the recovered cases. There were 34 operations for the radical cure of hernia, and of these 32 were successful. The anæsthetic table shows that chloroform was administered 1,210 times, ether 13 times; chloroform and ether 9 times; A.C.E. mixture 7 times; and ethyl chloride 13 times.

